# Dead end? Suicide as a crossroads of emotion dysregulation and illness burden

**DOI:** 10.1192/j.eurpsy.2026.10275

**Published:** 2026-07-31

**Authors:** P. Courtet

**Affiliations:** University of Montpellier, Montpellier, France

## Abstract

**Abstract:**

Suicide is often conceptualized as the tragic endpoint of psychiatric illness. However, accumulating evidence suggests that suicidal behavior emerges not merely from diagnostic categories, but from the dynamic convergence of emotional dysregulation and cumulative illness burden. This lecture proposes a crossroad model in which suicide risk reflects the interaction between proximal failures in affect regulation and distal biological and clinical load.

Emotion dysregulation constitutes a central proximal mechanism of suicidal crises. Heightened affective reactivity, impaired prefrontal control, cognitive constriction, and anhedonia converge to reduce the capacity to tolerate distress. Neurobiologically, alterations in fronto-limbic circuitry, stress-axis dysregulation, and impaired reward processing contribute to this vulnerability. Yet these mechanisms unfold within a broader context of illness burden.

Psychiatric comorbidity, chronic somatic disease, pain syndromes, and systemic low-grade inflammation progressively erode resilience. Biological stressors such as inflammatory activation, hypothalamic–pituitary–adrenal axis alterations, and potential blood–brain barrier dysfunction may lower the threshold at which emotional dysregulation translates into suicidal behavior. Illness burden may therefore act as an amplifier, transforming transient distress into existential crisis.

Integrating these dimensions allows a shift toward precision suicidology. Rather than treating suicide risk solely through categorical diagnoses, this framework emphasizes the identification of dominant vulnerability pathways—such as inflammatory profiles, reward-processing deficits, stress-sensitivity phenotypes, or severe emotion regulation impairments. Stratifying patients according to these interacting biological and psychological mechanisms may inform personalized preventive strategies and targeted interventions.

Suicide should not be viewed as a dead end, but as a systemic tipping point—where emotional collapse meets accumulated biological and clinical load. Advancing prevention requires bridging psychiatry, neuroscience, and somatic medicine to develop mechanism-based, individualized models of care.

**Disclosure of Interest:**

None Declared

